# Experiences of people from minoritised groups who report healthcare-related harm in the UK: a qualitative socioecological study exploring factors contributing to unsafe care

**DOI:** 10.1136/bmjoq-2025-003851

**Published:** 2026-02-06

**Authors:** Lavanya Thana, Helen Crocker, Shivali Modha, Linda Mulcahy, Ford Hickson, Catherine Pope, Charles Vincent, Helen Hogan, Michele Peters

**Affiliations:** 1 Department of Health Service Research and Policy, London School of Hygiene and Tropical Medicine, London, UK; 2 Nuffield Department of Population Health, University of Oxford, Oxford, UK; 3 UCL, Barnet, UK; 4 Centre for Socio-Legal Studies, University of Oxford, Oxford, UK; 5 Nuffield Department of Primary Care Health Sciences, Oxford University, Oxford, UK; 6 Department of Experimental Psychology, University of Oxford, Oxford, UK

**Keywords:** Patient safety, Social sciences, Health services research, Qualitative research

## Abstract

**Objectives:**

To capture the experiences of people from minoritised groups who self-report healthcare-related harm and their views on contributory factors to the harm.

**Design:**

In-depth one-to-one qualitative interviews, analysed using inductive and deductive methods to explore and then organise factors participants associated with healthcare-related harm and map these factors onto a socioecological framework (SEF).

**Setting:**

People from minoritised groups in the United Kingdom (UK) self-reporting harm arising from the National Health Service (NHS), recruited from community groups, social media and a survey of the general public.

**Participants:**

48 participants currently minoritised in the UK based on one or more of faith, ethnicity, disability, sexual orientation or gender modality who have experienced harm in the NHS.

**Results:**

Heterogeneous and interacting factors contribute to healthcare-related harms, spanning all five levels of the SEF: individual, interpersonal, community, organisational and societal. Multiple factors from powerlessness and low trust to unwelcoming NHS environments reinforce each other to increase risk of harm in minoritised populations. The SEF helped draw out less visible factors associated with the experience of unsafe care, including a health service designed around the needs of the majority population and societal attitudes to minoritised groups.

**Conclusions:**

Multiple individual factors are already known drivers of disparities in safety among minoritised groups such as language barriers and cultural differences in beliefs. The SEF enabled an expanded view of contributory factors to harm in these groups, thereby providing a wider set of potential interventions to address safety inequities. A narrow focus on improving the quality of interpersonal, relational care is unlikely to have a significant impact on safety improvement in minoritised groups without addressing structural and institutionalised processes that drive discrimination and exclusion.

**WHAT IS ALREADY KNOWN ON THIS TOPIC**

- People from minoritised groups experience differences in the safety and quality of healthcare.
- Research usually focuses on the experiences of one minoritised group and their individualised experiences rather than across multiple minoritised groups.

**WHAT THIS STUDY ADDS**

- By mapping experiences of people from different minoritised groups onto the socioecological framework, the study highlights the common factors driving healthcare safety in minoritised groups across individual, interpersonal, community, organisational and societal levels.

**HOW THIS STUDY MIGHT AFFECT RESEARCH, PRACTICE OR POLICY**

- To build an inclusive, equitable, high-quality health service for all, diverse less-often-heard voices need to be taken into account.
- Fundamental drivers of inequality are important to consider when implementing changes in the National Health Service to make it a safe space for everyone.

## Introduction

 People receiving healthcare are ‘privileged witnesses of events’, being at the centre of treatment and able to observe the whole process of care.^1^ However, their unique perspectives on safety are undervalued and underused,^2 3^ particularly for individuals from minoritised groups, who are often socioeconomically disadvantaged and whose voices tend to be sidelined within society.^4^ These groups face disparities in patient safety that are relatively underexplored.^5^,^7^ Addressing these inequities is critical as harm is associated with long-term negative health outcomes and reduced trust in, and future use of health services.^8 9^ Existing studies of safety disparities focus on single groups (eg, ethnic minority or people with a disability), specific aspects of care or specific outcomes.^710^,^12^ Less is known about factors that are common across minoritised groups.

In the UK, the National Health Service (NHS) is constitutionally obliged to reduce health inequalities, including ensuring good quality, safe care for all.^13 14^ The current NHS Patient Safety Strategy states: “The NHS does not yet know enough about how the interplay of normal human behaviour and systems determines patient safety”.^15^ Individuals’ behaviours, experiences and outcomes, including health, are shaped by multiple layers of influence, not just individual choices. The socioecological framework (SEF) is valuable for illuminating how people live within systems (individual, interpersonal, community, organisational and societal) and how these systems interact.^16^ The SEF can highlight how a complex interplay of social factors at different system levels interacts to produce safety-related behaviours, events and outcomes. While the SEF has proved valuable to analyse factors from single minoritised groups, it has rarely been used to examine factors related to unsafe care that are common across different minoritised groups.

This study aimed to capture harm experiences of people from diverse minoritised groups, who self-reported harm by treatment or care or lack of access to it, and their perspectives on factors contributing to unsafe care. We mapped these contributory factors onto the SEF to show their span across societal levels.

## Methods

### Overview and context of the research

**Figure 1:**
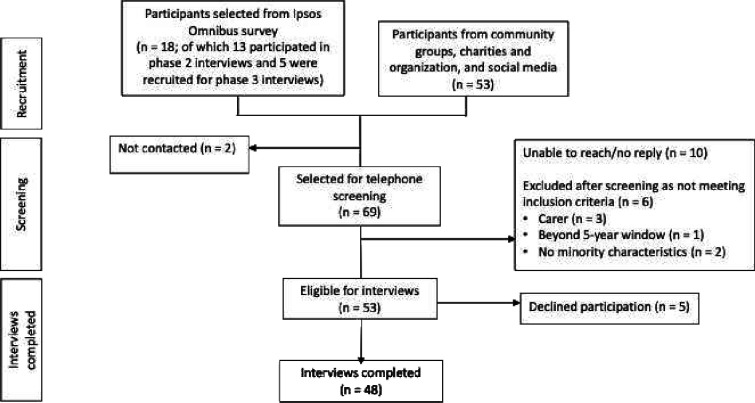
Participant recruitment.

This interview study was part of a mixed-methods study that included: (1) a UK-based cross-sectional general population survey (10 082 respondents) capturing the frequency and impact of harm and actions taken after harm^17^; (2) qualitative interviews with 49 survey participants further exploring factors influencing actions after harm^18^ and (3) qualitative interviews (undertaken in 2022 and 2023), reported in this paper, with people from minoritised groups recruited from the survey and through community networks and social media (figure 1).

### Sampling and selection criteria

The survey sample was generated from those reporting harm (9.7%, n=988) that consented to being contacted for interview and by sociodemographic factors. Community recruitment included contacting potential interviewees via faith groups, user-led health-related charities and support groups, health and social care-related networks, and safety advocacy groups including Action Against Medical Accidents. Notice of the study was posted on web-based forums and social media (via X (Twitter), LinkedIn and Facebook). Purposive sampling ensured maximum diversity across ethnicity, faith, disability, sexual orientation and gender with a target sample of 10–12 people per group. Potential participants recruited through community and social media underwent a short telephone screening interview to collect similar information to the survey, establish eligibility and select participants from minoritised groups under-represented in the survey sample.

An expert advisory group (EAG) consisted of patient and public involvement members, representatives from safety advocacy groups, clinicians and legal professionals. The EAG helped develop recruitment materials, signposted researchers to their contacts within community and advocacy groups and charities and contributed to the analysis and interpretation.

### Data collection

The topic guide was developed from the academic literature, with input from academic experts and the EAG. In-depth narrative-style interviews lasting 60–120 min were conducted virtually by LT and HC and audio recorded on a secure encrypted device. The interviews explored experiences of care-related harm, subsequent thoughts and actions, factors contributing to unsafe care and how factors related to minority characteristics might contribute to the risk of harm (topic guide online supplemental box S1, online supplemental material). All participants gave informed written consent.

Given the challenging nature of the topic, safeguarding measures were taken including inviting participants to have a family member or friend present for the interview and being clear that they were free to stop the interview, reschedule or withdraw at any point. Those not proficient in spoken English or with a hearing impairment were offered a supported interview with an accompanying friend or family member or a language or British Sign Language interpreter. After the interview, participants received information on organisations providing support for those experiencing healthcare-related harm and a thank you voucher.

### Analysis

The analysis focused on identifying factors that increased vulnerability to healthcare-related harm. The interview recordings were transcribed by a professional transcription company and checked by LT and HC for accuracy. Inductive analysis using a systematic approach adapted from Constructionist Grounded Theory was undertaken^19^ to enable understanding of the interactions between people and societal phenomena such as groups, institutions and culture.^20^ Following line-by-line coding of the transcripts of early transcripts, LT generated an initial coding framework. A quarter of transcripts were coded by CP, HH, and MP to critically evaluate and add to the coding framework. The team remained critical of the analysis throughout, open to reconfiguring or discarding categories as the analysis progressed. Interim findings were presented to, and commented on by, the EAG and wider study team as a further credibility check.

As data collection progressed, the categories and their relationships were iteratively mapped by LT alongside development of analytic memos. The inductively developed categories were then mapped to the five levels of the SEF (based on McLeroy *et al* (table 1)^16^); individual, interpersonal, community, organisational and societal (by LT, HH and MP) using an interpretative approach.

**Table 1:** Descriptors of the five levels in the socioecological framework

Individual	Personal characteristics that can influence behaviours including knowledge, attitudes, self-efficacy, gender, age, beliefs and capabilities
Interpersonal	Relationships, social networks and support systems comprising friends, family, colleagues and others that can influence behaviours including the quality of relationships, communication patterns and social norms
Community	The environment in which people live, including social norms and cultural influences and resources within those communities
Organisational	Organisations with practices, policies and environments that provide a service or services
Societal	Structural, political and economic systems including local, national and international laws and policies that influence health across populations

Adapted from McLeroy *et al*.^16^

## Results

### Participants

Interviews were conducted with 48 people from minoritised groups across Great Britain who self-reported healthcare-related harm from NHS treatment or lack of treatment in the past 5 years. 18 participants were recruited via the survey (designated by identifier ‘1’ before the participant number), 13 of whom had completed interviews in phases 2 and 5 were recruited for phase 3. 35 participants were recruited through community groups and social media (designated by ‘2’). 30 were female, 44 under 60 years, 33 had a disability, 33 were from ethnic minoritised backgrounds, 11 indicated a sexual orientation other than heterosexual and 4 were not cisgender (table 2). The majority reported multiple minoritised characteristics and just over one-third were socially disadvantaged (annual household income less than £20 000 or in receipt of benefit payment) (table 2). The majority reported moderate or severe physical or emotional impact from harm.

**Table 2:** Participant characteristics (n=48)

		Frequency (n)
	Female	30
Sex registered at birth	Male	18
Age	18–24	8
	25–34	21
	35–44	10
	45–54	6
	55–59	1
	60–64	1
	65+	1
Ethnicity	Arab	2
	Asian Chinese/British Chinese	3
	Asian Indian/Pakistani/British Indian/Pakistani/Bangladeshi	13
	Black Caribbean/black British/black other	9
	Mixed background	3
	Other	2
	White British/Irish	15
	White other	1
Faith	Agnostic	2
	Christian	8
	Hindu	5
	Muslim	8
	No faith	8
	Quaker	1
	Sikh	1
	Declined to answer	1
	Missing data	14
Gender identity	Trans man/woman	2
	Non-binary	1
	Questioning identity	1
	Cisgender	44
Sexual orientation	Asexual	2
	Bisexual	2
	Demigray asexual	1
	Gay	5
	Pansexual	1
	Heterosexual	23
	Missing data	14
Disability	Yes	33
	No	7
	Missing data	8
Number of minority characteristics		
(ethnicity, faith, disability, sexual orientation, gender)	1	17
	2	10
	3	14
	4	7
Socioeconomic disadvantage	<£20 000 annual household income or on benefits	17
Highest educational or professional qualification	No formal qualifications	1
	Vocational qualifications	6
	Secondary education to 16 years of age	5
	A-level or equivalent (usually up to 18 years of age)	7
	Bachelor's degree or equivalent	17
	Masters/ PhD or equivalent	9
	Missing data	3
Employment status	Full-time student	3
	Full-time paid work (30+ hours per week)	7
	Part-time paid work (8–29 hours per week)	7
	Not in paid work due to long-term illness or disability	20
	Retired	2
	Self-employed	5
	Unemployed and seeking work	4
Annual household income	In receipt of universal credit	13
	Less than £10 000	2
	£10 000–£20 000	2
	£20 001–£30 000	5
	£30 001–£40 000	7
	£40 001–£50 000	4
	£50 001–£60 000	1
	£60 001–£70 000	2
	More than £70 000	2
	Missing data	10
Region	East Midlands	4
	Eastern	2
	London	13
	North West	3
	Scotland	2
	South East	10
	South West	2
	Wales	1
	West Midlands	7
	Yorkshire and Humber	4
Physical harm	No impact	3
	Mild	5
	Moderate	23
	Severe	16
	Don’t know	1
Psychological harm	Mild	2
	Moderate	17
	Severe	29

### Experiences of harm

Participants reported a range of physical and emotional harms resulting from diverse causes, including lack of access or delays in receiving treatments or care, poor care coordination, missed or delayed diagnoses and poor inter-relational care, involving stereotyping and discrimination (see table 3). They commonly reported an initial struggle to come to terms with what had happened, followed by a search for explanations and support. Many felt ignored and rejected in their search for answers, which resulted in feelings of anger, hurt, dejection and a sense, for some, of being abandoned.

**Table 3:** Examples of unsafe care among participants

Participant characteristics (E=ethnic group, F=faith group, LI= low income ie, less than £20 000 per annum, D=disability, S=sexual orientation)	Examples of unsafe care
2 P034: E, F, LI (self-assessed as mild physical and moderate psychological impact)	Participant attended the Emergency Department with foot injury and in severe pain. They could see that there was a broken bone on the X-ray but were dismissed by the doctor and discharged with inadequate ankle support and incorrect advice related to mobilisation. The participant felt strongly that they had been inappropriately dismissed because their pain or needs were not conveyed in a way that the doctor would recognise. The participant continued to have pain and later required referral to the orthopaedic team for further investigation and treatment.
1 P040: E (self-assessed as severe physical and psychological impact)	The participant developed pain in the thumb due to repetitive movements at work. Initially, the GP prescribed anti-inflammatories and rest. The pain did not settle and the patient visited the GP on several occasions but was dismissed as having ‘normal pain’ which needed no further action. Eventually, they had to give up their job because of the pain. Subsequently, on seeking advice abroad, scans showed inflammatory damage to the bone. Returning home, on a further visit to primary care, the participant saw a different GP who took note of the scan findings and made a referral to an orthopaedic surgeon. After a further year on an NHS waiting list, an operation was undertaken, 3 years after the initial presentation. This helped reduce the pain, but the participant was left with restricted movement in the hand.
2 P026: E, R, D, LI (self-assessed as severe physical and psychological impact)	This participant is neurodiverse and known to have blood cancer. Their hospital team failed to follow the protocol for high-dose methotrexate administration, which led to the participant’s kidney injury, some of which was not reversible. There was a delay in identifying the condition and instituting remedial treatment. Despite the participant’s knowledge of what needed to happen and their raising of concerns, they were continually dismissed and were provided with factually wrong information. The kidney damage resulted in a higher risk of harm from future chemotherapy treatments.
2 P002: D, LI (self-assessed as severe psychological impact)	The participant had cerebral palsy from birth, uses a wheelchair and requires assistance with tasks of daily living. They developed an acute mental health crisis which the community Crisis Resolution Team could not manage as the participant did not wish to take recommended medication. When their condition worsened and they began to feel suicidal, their family doctor requested admission at a local psychiatric inpatient facility. No accessible bed was available. Another hospital was identified with a bed but this admission was cancelled because the participant was in the wrong geographical catchment area to be funded. Eventually, they were able to engage a therapist using a personal health budget provided by the area health board. The lack of access to timely treatment prolonged suffering and delayed recovery.
2 P023: E, F, S, LI (self-assessed as moderate physical and severe psychological impact)	Due to severe mental illness, the participant was sectioned under the Mental Health Act and had a 6-month admission to a psychiatric hospital. They were forcibly restrained on a number of occasions, causing multiple physical injuries including a wrist fracture. The ward environment felt extremely unsafe, not only because of the participants fear of staff but also the frequent violence between other patients and staff and sometimes between patients. The participant was constantly in fear of being attacked because of their ethnicity or sexual orientation. Their ability to seek comfort in spiritual practices was limited by inadequate facilities, and when this was mentioned to the psychiatrist, they were told that there was no need to follow these practices while in hospital. Appeals to ward managers and psychiatrists over mistreatment were dismissed. The participant felt stereotyped because of their background and felt they did not get the support they needed to heal, which was in part due to staff not making an effort to understand some of the factors precipitating their illness.
2 P012: E, F, D (self-assessed as severe physical and severe psychological impact)	The participant's concerns were continuously dismissed and they were made to feel like a nuisance. This led to a delay in the diagnosis of a throat cancer and the need for more extensive surgery than if it had been picked up earlier. Not only were they left in physical and psychological pain after treatment, but they could no longer continue in her job, losing financial independence and having to rely on her husband.

GP, general practitioner; NHS, National Health Service.

### Contributory factors to unsafe care

Below, we present contributory factors to unsafe care at each SEF level. This approach is not intended to diminish the complex interactivity of the factors involved but rather to highlight factors across minoritised groups that contributed to this complexity. For most participants, multiple contributory factors to unsafe care were found at each SEF level and across levels which acted in concert to compound and reinforce the risk of physical or psychological healthcare-related harm.

### Individual factors

Participants described how personal, family or community experiences of poor care in the past reduced trust and increased fears of not being listened to, fair treatment or access to treatment during NHS contacts. A sense of personal powerlessness could create anxiety that unwanted or inappropriate treatments would be forced on them. These negative feelings often engendered a sense of alienation and the need to remain alert to the possibility of being harmed. This was reinforced when participants encountered difficulties understanding what was going on due to the use of overly medical language or technical terms by healthcare professionals (HCPs). Some participants reported how concerns about stigmatisation and privacy could lead them to hide their minority characteristic during consultations, if this was possible.

So I basically, whenever, I always test out my LGBT identities, I always, before I mention that I have a trans ID I will mention I have a wife. It just, for me that kind of buffers and it tells me that if they’re a bit funny with me because I’m seemingly gay, then I know that, actually, it’s not a safe space for me to be, to be outing myself as trans. [The participant goes on to say] …I’ve had past experiences where I’ve been laughed at by doctors for being trans….so I didn’t, I didn’t disclose. So I was very worried that I wouldn't get the care that I needed or that the care that I was receiving would get worse. 2 P019

Others chose to avoid healthcare, especially where cultural norms made family care, home remedies or use of faith or traditional healers valid options. Some sought healthcare abroad. One participant was able to confirm the severity of her condition only after diagnostic tests were undertaken abroad, which subsequently led to more intensive treatment in the UK, but not before permanent damage had been sustained due to delays.

after one month, or after six weeks, that’s when I contacted my dad [in home country] and said this is the case, and the GP here thinks it’s normal, but I think there was some underlying cause. I need to be checked more but here I can’t get the emergency services……That was the final time when I talked to dad, then my dad made me, he made an appointment with the musculoskeletal doctor in [home country]. 1 P040

Participants described how together these concerns often increased reluctance to seek medical help when needed.

### Interpersonal factors

Poor relational care reinforced low levels of trust and the sense of alienation. Experience of stereotyping was common with one individual describing a HCP falsely attributing psychological symptoms to an arranged marriage. Such behaviour could lead to inadequate exploration of wider diagnostic options, inappropriate management decisions and treatments that participants were less inclined to believe would be effective. The general sense was that HCPs frequently dismissed symptoms, particularly in relation to pain, with pain scores being challenged or downgraded. Others reported enduring deterioration and making multiple clinic visits before their concerns were taken seriously and acted on.

it was all because I didn’t convey pain in the way that they wanted me to convey pain and so I was not immobilised, like I walked, I was given a soft boot and walked on that and although later on I did get physio, the MRI and so on and the orthopaedic referral, that was not given to me in the first instance because I didn’t convey pain the way that they wanted me to basically. 2 P034

Participants described continuously needing to justify why they required support and being treated in a way that suggested that they or their needs were a nuisance or a burden. Others described changing their behaviour to conform with system expectations to access the care they needed, playing down some needs or choosing not to question HCP decisions. Feeling ‘not known’ by HCPs meant that participants’ capacity to engage in decision-making or self-management was frequently underestimated. Some felt it was a continuous battle to be seen and heard.

I’d already asked them to put on my notes like an urgent thing that they saw straightaway to say that I had cerebral palsy and my speech is unclear, so that should have been on the screen straightaway but this crisis worker said “I’m not speaking to you until you sober up” and it was like 10 am in the morning and I was really distressed by that and tried to say “I’m not drunk, I have cerebral palsy” but they’d already put the phone down on me. 2 P002

Support by family or friends to navigate the healthcare system or to act as a buffer to safety risks varied. Language and cultural differences often acted as a barrier, as well as cultural norms of stoicism and reluctance to question HCPs. At its most extreme, participants were pressurised to remain silent.

when he would say that you’re fine and he wouldn’t give me anything more than Ibuprofen, my family wouldn’t take me seriously as well. They would say that maybe you are just feeling that way but obviously he’s a doctor and if there was something serious he should have picked that up. I was sad that actually—I felt like misguided, and nobody was believing me, what kind of pain I’m experiencing. 1 P040

### Community factors contributing to unsafe care

Negative perceptions of NHS services and HCPs held within communities shaped individuals’ attitudes and perpetuated distrust and reluctance to use services. When asked about community perceptions of healthcare, one participant responded:

I think on the whole poor…I think there’s a lot of barriers to healthcare which increases a lot if you’re in any minority group really and then if you’re a combination [of minority groups] even more so. And I know in my friends who are of colour and they’re trans, I have a particular friend who is black, non-binary but female presenting and young, and the combination of all of those is they’re never listened to, like we talk about it quite a lot and it’s just…we need to vent about generally how rubbish it is, so you’ve got all the barriers to healthcare anyway and then when you’re actually in the healthcare all the barriers and the things that are inaccessible or non-inclusive. I kind of feel I’m at a point where I don’t want to access healthcare because it, I’m either like not listened to or I’m dismissed. 2 P035

Prevalent community attitudes included a sense of powerlessness, a passive acceptance of unsafe care and reluctance to speak up about harm. Some participants described how acceptance of the status quo could be reinforced by local faith leaders and community members.

I had intention to lay complaint, that’s after leaving the ward, but then I think when I had conversations with my faith leader, I was just told to forget the whole incident and then move on, focused about the life that I have now, and be happy about it. 2 P017

Minoritised groups with rights-based community networks (eg, disability groups) had increased awareness of NHS obligations to meet their specific care needs and the process for complaining if needs were not met. Such participants were more likely to feel empowered to positively influence their healthcare safety.

### Organisational factors contributing to unsafe care

Organisations consist of environments, policies and practices that can each impact on safety. Participants described how not seeing their identities mirrored in the NHS workforce or represented in key symbols increased their sense of being an outsider. The perception of the NHS as ‘one size fits all’, catering to the majority, suggested to participants that they should try to conform. Their feelings of exclusion created anxiety as to whether HCPs would be respectful and unbiased and increased concerns over sharing personal information with HCPs. Many described experiences of discrimination by staff or other patients.

Now what really happened specifically was I was not being attended to with necessary care and compassion, not…, and I was in a state where I needed more than just physical care, but I needed to feel emotionally supported…Yeah, because the hospital attendants were white, they were white. Coloured people, and I’m Caribbean, and I can feel that disparity. I could feel that disparity in the kind of attention they give to other [white]patients in the ward, and how they attended to me. The other patients who had less physical condition were attended to with more enthusiasm, and more empathy than I was. 2 P017

You know, there are instances of overt homophobia in the waiting room, that was really difficult, by another patient and their husband and it was just, and it was very, it was very unsafe and I don’t think anyone truly realises that, actually, you’re vulnerable to begin with in a hospital situation… before you enter spaces that, where people, don’t think or they just don’t care. 2 P019

Some acknowledged that these were systemic failures rather than due to individual HCPs. Poorly resourced interpreting services in both primary and secondary care also impacted safe care. Family or friends were often used as interpreters, severely hindering privacy and independence. One person with a disability was denied hospitalisation during a mental health crisis due to the lack of a disability-adapted room. The absence of basic resources needed to meet care needs was most acutely felt by participants with disabilities.

### Wider societal factors

Discrimination in access to services and within the care experience was the most frequently mentioned societal issue associated with unsafe care. Some participants referred to this as a reality that they had learnt to accept.

Every time, the minute you walk in and you’re Chinese, at the time, they will—it feels like they put you in a bracket straight away, that it’s not urgent, it’s not important, that we’re not—we’re just sponging off the NHS. Well, yes, we accept the stuff quite—I know that it’s bad, we shouldn’t accept it, but we’ve kind of grown up and accepted how we get treated. 1 P041

Although some participants could not point to specific outright discrimination, they often experienced bias and others receiving better quality treatment. Participants felt that biases stemmed from wider societal perceptions of their communities that filtered through into healthcare contexts.

I just don’t feel a part of the NHS… Because I don’t want to be in the company of most of the doctors in the first place. I don’t feel like there’s enough care in a way or understanding. 1 P008

## Discussion

**Figure 2:**
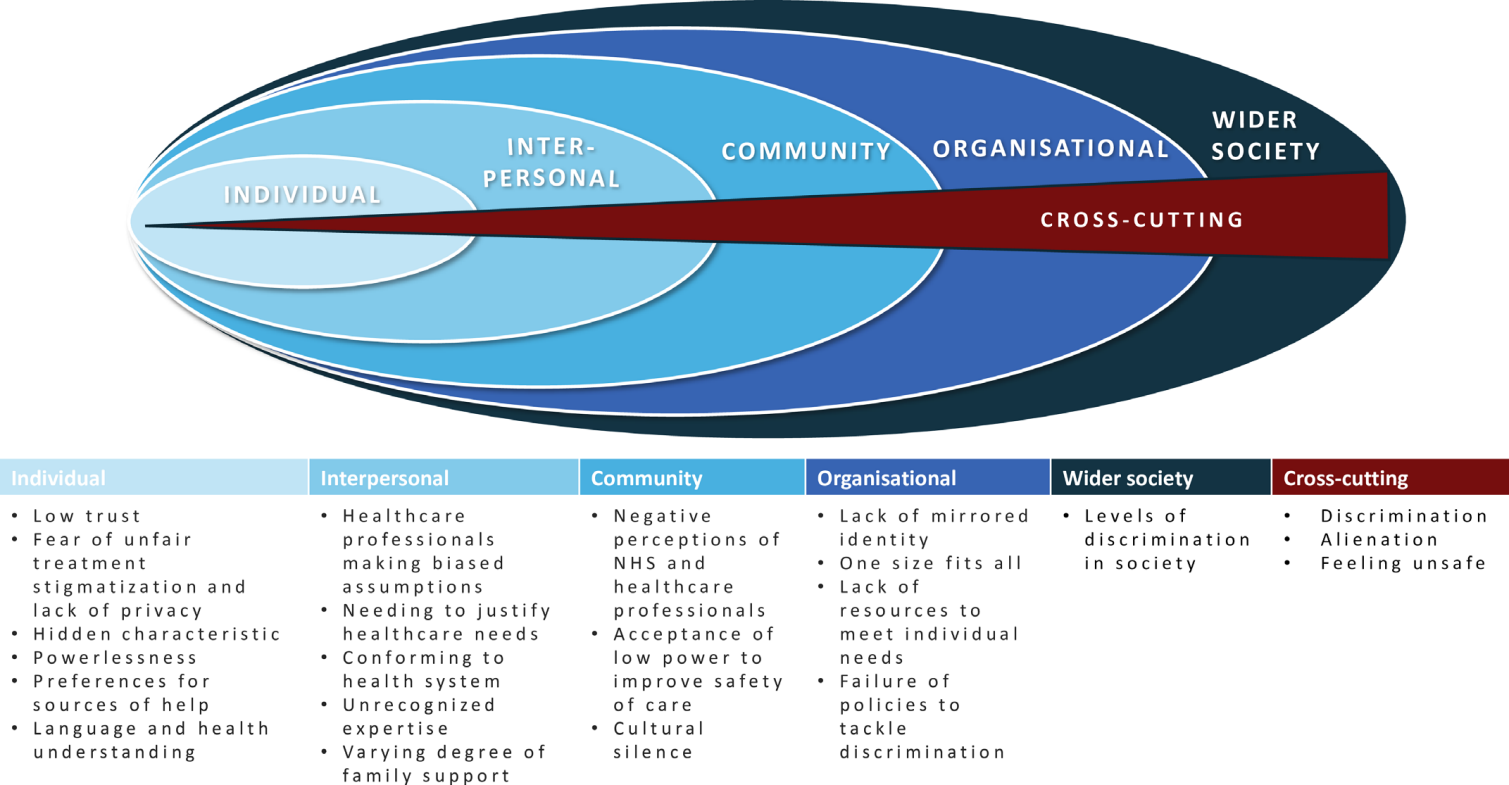
Socioecological model of contributory factors for patient safety among minoritised groups.

Our study shows the complex web of contributory factors that have the potential to increase the risks of healthcare-related harm for people from minoritised groups. Mapping these factors onto the SEF highlights their distribution at individual, interpersonal, community, organisation and societal levels, as well as cross-cutting factors (figure 2). Many are already known drivers of safety disparities in their own right.^7 21^ Our analysis builds on this research by demonstrating how individual factors are interconnected and highlighting their commonality across minoritised groups.

Typically, research into safety inequities has focused on contributory factors at individual and interpersonal levels and echoes our findings. Language proficiency and health literacy are commonly cited as increasing risk of harm.^22 23^ Poor relational care excludes the patient from the consultation, the diagnostic process and ongoing management and precludes safe, effective and inclusive healthcare practices that maintain trust and prevent ‘exiting’ from the health system^24^ contributing to delayed or missed diagnoses, failures to stabilise ongoing conditions and lost opportunities for preventative interventions.^25^

Our findings highlight how stereotyping and implicit and explicit bias are important underlying contributors to poor relational care. Stereotyping can lead to HCPs foreshortening the assessment of symptoms, making presumptions about the capacity of patients to understand or manage their own conditions, reducing discussion of options for care and under use shared decision-making.^26 27^ Despite the NHS having a diverse workforce, HCPs are part of society and reflect society’s prevalent views and biases in relation to minority groups.^28^ Also, HCPs work in organisations that are often not effectively designed to meet the social, cultural or linguistic needs of minoritised groups and, as such, are experienced as ‘one size fits all’ and exclusionary by these patients.^29 30^ Observation of the majority population receiving a different standard of care or experiencing the failure of staff to call out discrimination reinforces the views of some minoritised individuals that the NHS does not serve them or their communities.^31 32^

Societal disadvantage, community attitudes to the NHS and previous poor health experiences lower expectations of good quality and safe care for marginalised populations even before entering the NHS.^32^,^34^ These factors impact on trust and create doubt about fair treatment and heighten fears about not being kept ‘safe’. For minoritised individuals and communities, who may already feel stigma from society, interactions with health services can be profoundly disempowering and alienating, not only contributing to increased risks of physical and emotional harm but also reducing willingness to report the harm afterwards.^35^ These undocumented harms can have little influence on safety improvement initiatives.

Feeling unsafe within health services was a common complaint across all groups in our study and often the result of disrespectful or dismissive behaviour by HCPs. These behaviours are also commonly experienced by non-minoritised patients and are often considered ‘poor patient experience’ rather than a safety threat.^7 36 37^ However, their contribution to ‘feeling unsafe’ seems to have a larger part to play in harm in people from minoritised groups, particularly when compounded by wider contributory factors at community, organisational and societal levels. The majority of study participants reported both physical and emotional harm and most commonly self-rated their impact as moderate or severe, which may reflect how experiencing both emotional and physical harms potentiate each other, leading to poorer outcomes overall. To date, relatively little attention has been given to this area in safety research, but given the potential for reducing trust and interfering with engagement, its likely contribution to safety inequities may be substantial.^36^,^38^

### Implications for practice, policy and research

Mapping our findings onto the SEF draws attention not only to the wide array of factors contributing to safety in minoritised groups but also to a more diverse set of potential improvement opportunities to help tackle safety inequities.^6 21^ Building a safe and inclusive health service for all users requires moving beyond single interventions such as cultural sensitivity, which on their own, increase attention on individual and group characteristics pulling focus away from interventions such as reducing barriers to care or improving quality that might address factors influencing safety held in common across minoritised groups.^39^ Like members of majorities, people from minoritised groups want a person-centred approach that treats them with empathy and respect.^40^ They want to be recognised for their expertise and be more effectively engaged in shared decision-making.^41^ Enabling clinicians to provide such care is a critical building block of safety alongside provision of professional interpreters and patient advocates.^42^,^44^

Healthcare organisational leaders are responsible for fostering a just, equitable and safe culture of care.^45^ Developing an organisation with a diverse workforce that mirrors local populations, that puts the patient perspective at the heart of safety and provides services tailored to needs is important contributors to such a culture.^6 46^ Engagement and listening to the voices of minoritised and economically challenged populations is essential.^47^ Collaborative service design and evaluation alongside providing opportunities for involvement of these populations in training HCPs will not only benefit the NHS but could also play an important role in reducing negative perceptions of the NHS among key minoritised groups.^40 46 48^

Health systems such as the NHS have a duty to address inequalities in health.^13 14^ Diversity, equality and inclusion are integral components of improving the quality and safety of care and require examination of structural and institutionalised processes that drive discrimination and exclusion.^49^ Attention will also need to be paid to capturing data to monitor progress. Policy and service development to improve safety and address inequalities will need better discrete data on subpopulations’ safety including trends in prevalence of harm and the nature of harm experiences.^21^ One major contributor to our understanding of the scale and scope of harm is patient safety incident reporting systems. These clinician-focused systems rarely capture the voice of patients and families, and the contribution from marginalised populations is likely to be even less.^50 51^ Thought needs to go into how such systems define harm to ensure recording of not just physical harms but also failings in psychosocial aspects of care that are likely to have a disproportionate impact on minoritised populations.^52 53^ Systems-based, equity-focused approaches to determining contributory factors will also be needed.^54^ Ultimately, wider sources of information on harm that better capture contributions from minoritised people will be needed.^55^,^57^ Such changes require national prioritisation and incorporation into NHS strategies to improve safety.

Although there is a growing body of research capturing the burden of harm in discrete subpopulations,^7 10 12^ more work needs to be directed at how intersectionality (the interaction between an individual or group’s different characteristics such as age, gender, sexuality, disability and/or socioeconomic status) increases risk of harm and poor outcomes.^58^ The majority of study participants in this study had more than one minoritised characteristic. As well as multidisciplinary approaches to enable a better understanding of the mechanisms that maintain safety inequity, research could also help elucidate why some groups can become empowered (eg, those with disability) and find a voice within the system to get their needs met. Knowledge of these resilience factors could help guide safer service design. Finally, addressing safety inequities requires the development of more patient-centred safety indicators that measure aspects of safety such as trust, respect, continuity and involvement, which are important to patient’s and their families, particularly those from more marginalised communities.^36 59^

### Strengths and limitations

Capturing the perspectives of people who use healthcare services contributes to creating a whole system view of safety and supplements information gathered by other approaches. Our study included diverse people with minoritised characteristics and vulnerable socioeconomic status whose voices are less likely to be included in research and who may, therefore, provide additional unique insights on how to improve healthcare quality and safety. Participants were grounded in their own experiences of harm and therefore carried authenticity when speaking about healthcare safety. To strengthen our inclusive approach, harm was self-reported, with no restrictions on the types of harm but aimed to include emotional harm which may be more common in minoritised populations.^1^ We also took an inclusive approach to our analysis by highlighting commonalities across minoritised groups which amplifies their collective voice and gives important insights on how to build a more inclusive health service.

A potential limitation is recall bias as participants’ accounts were retrospective. We accounted for this by limiting harm experiences to 5 years prior to the study. Another potential limitation may be that the study relied on participants being able to identify and convey pertinent issues, some of which are ingrained in societal norms and organisational structures, and likely to be relatively invisible at individual or interpersonal levels. We attempted to account for this by asking specific questions around topics such as discrimination and employing the SEF in our interpretive analysis. Finally, we sampled from a limited number of minority groups and our participants were predominantly under 60 years old; therefore, all relevant contributory factors might not be represented.

## Conclusions

The NHS has a duty to address health inequalities and to provide safe and high-quality care for all. The drivers of inequities in safety have been relatively underexplored, and highlighting the experiences of diverse people who are less heard is key to identifying and addressing inequities. It also requires examination of not only individual and interpersonal factors that drive differences but also the structural and institutionalised processes that compound them, including discrimination and exclusion. Addressing disparities in safety among minoritised groups will require national action at multiple societal levels including incorporation, as a priority, into NHS strategies toF improve safety.

## Supplementary material

10.1136/bmjoq-2025-003851 online supplemental file 1

## Data Availability

No data are available.
