# Supplementary material for: Experiences of people from minoritised groups who report healthcare-related harm in the UK: a qualitative socioecological study exploring factors contributing to unsafe care
Source: BMJ Open Qual. 2026 Feb 6;15(1):e003851. doi: 10.1136/bmjoq-2025-003851 (PMC12887519; doi:10.1136/bmjoq-2025-003851)
Supplement: online supplemental file 1 [file bmjoq-15-1-s001.docx]

## **Supplementary File:** Experiences of people from minoritized groups who report healthcare-related harm in the UK: a qualitative study using the socio-ecological model to explore factors contributing to safety

## Additional questions added to topic guide to further explore minority group experiences are highlighted.

Box S1: Qualitative Interview Topic Guide

1. **Introduction**
2. **Opening question**

*(Aim: Main participant narration to understand their adverse experience and any aftercare received. Interviewer should listen attentively without interrupting the participant’s account.)*

Example: “I am interested in understanding more about your experience of the treatment or care you received [where possible, using the participant’s own terminology as used during pre-interview call]. Please tell me about this in your own words.”

1. **Research topics**

*(Aim: Questioning phase to explore and expand on pertinent aspects of the respondent’s experience, as well as their decision to take any further action, that have not already been conveyed in the participant’s own organic story-telling.*

1. Pre-treatment

- Prior experience of healthcare (e.g. Did the participant have any previous adverse experiences in health settings? If so, did this have any influence on their experience of the treatment or care they received on this occasion, as well as on the decision to make a claim? Did the participant have any previous positive experiences of healthcare? If so, in what ways did this experience contrast with their experience of harm? How, if at all, did the feeling of ‘safety’, the lack of it, or feeling ‘unsafe’ contribute to their experiences of healthcare?)
- Participant’s circumstances at time of the illness or event that required treatment or care. (e.g. What caused them to seek treatment?)
- Pre-treatment thoughts, feelings, and expectations about the treatment. (e.g. What were they hoping would be the outcome of treatment? Were these expectations met? Were these aligned with the clinician’s expectations?)

1. Experience of care/treatment

- Treatment and care received during harmful experience (e.g. side effects, psychological harm, medical injury or harm, worsening condition)
- Outcome of healthcare experience/treatment (e.g. What was the physical impact? How did this experience or outcome affect them emotionally and psychologically?
- And in their usual activities and environment? How long did these consequences last?)
- How was this incident or outcome handled by the service/staff involved in care (e.g. Was treating clinician’s view aligned with participant’s own view of the outcome? Was an explanation for the outcome provided?)
- Treatment relationship with clinicians, nurses and other staff involved in care (e.g. What were the participant’s feelings toward staff? To what extent did they have confidence in their ability to provide the treatment required? How if at all did your relationship with staff change over the course of your experience?)
- Personal factors that might have influenced treatment and experience of healthcare (e.g. Some people say they have been treated differently by staff in healthcare settings, are you aware of this happening?) Have you had any experiences such as this? Was your healthcare provider aware of your [ethnicity, religion, etc.]?, How did your healthcare provider become aware of your [ethnicity, religion, etc.]? How, if at all, did your [ethnicity/religion/sexual orientation/gender identity/disability] play a role in your healthcare experience? How, if at all, did your healthcare provider’s knowledge of your [ethnicity/religion/sexual orientation/gender identity/disability] influence the treatment or care that you received?) Have you ever experienced discrimination in a healthcare setting because of your [ethnicity/religion/sexual orientation/gender identity/disability]?
- Environment outside of healthcare setting that might have influenced experience of care (e.g. work/study, family and support networks, housing, financial circumstances)

1. Post-adverse experience/injury

- Any disclosure of concerns about care or outcome by or on behalf of the person in receipt of treatment to the healthcare practitioners involved in care, service, external practitioners, other organisation(s), friends/family, using online platforms
- How the service/healthcare practitioners involved in care/others responded to incident (e.g. Their reactions, attitudes and clarity of communication, and whether an apology was given/issued) and how the participant felt about this response
- Further treatment to aid recovery from any injury and/or negative psychological effects (e.g. What, if anything, was offered? Was it helpful? What was expected? Were there other resources that helped/hindered their recovery?)
- Personal factors that might have influenced ability to manage the negative impact of the harmful experience in the aftermath (e.g. How if at all has your [religious/spiritual/cultural/personal beliefs or identity] helped you following your experience? How if at all has it hindered things? Has your [ethnic/religious/sexual/gender] identity impacted your ability/willingness to seek treatment in the past (e.g. delayed or avoided seeking further help)? In what way has it had an impact?)
- Reasons behind decision not to pursue (e.g. Was further action considered at the time of the incident or subsequently? If so, what factors, personal beliefs, or other people in your life influenced the decision not to pursue this further? If participant had wished to, would they have known who to contact or how to go about making a complaint?)

1. Decision-making around informal or formal action

*[If not already known, find out whether the participant made a formal complaint or took legal action.]*

*IF* the participant chose not to take further action:

- Reasons behind decision not to pursue (e.g. Was further action considered at the time of the incident or subsequently? If so, what factors, personal beliefs, or other people in your life influenced the decision not to pursue this further? If participant had wished to, would they have known who to contact or how to go about making a complaint?)
- Other independently taken action (e.g. support sought from friends, family, community peers, religious groups, other clinicians than those involved in their care during the harmful experience, and other sources, such as professional counselling or charities; sharing experiences on social media or posting reviews online.)

*IF* the participant chose to make a formal complaint:

- Personal or external factors motivating decision to pursue complaint (e.g. altruistic reasons, seeking explanation and/or accountability, grief and associated emotions, family members’ influence, community peers, healthcare staff suggesting appropriateness of action)
- How was this dealt with by the service/organisation/treating clinicians (e.g. Adequacy of response? Was it dealt with in a timely manner?)
- Outcome of the complaint
- Any consequences and whether it aided recovery from harmful experience (e.g. How did they feel about the outcome of the complaint and how it was handled?)

*IF* the participant chose to pursue legal recourse:

- Personal or external factors motivating decision to pursue legal recourse (e.g. altruistic reasons, seeking explanation and/or accountability, grief and associated emotions, financial reasons, advertising, their own or other family members’ influence, community peers, healthcare staff suggesting appropriateness of action, advice received from legal professionals regarding whether or not to make a claim.)
- How was this dealt with by the legal and other professionals involved (e.g. Adequacy of case management? Was it dealt with in a timely manner? Etc.)
- How was the case settled? (e.g. financial compensation/no compensation, apology issued, etc.)
- Any consequences and whether it aided recovery (e.g. How did they feel about the outcome and how it was handled?)

1. Future perspectives about seeking treatment in a health services

- Undergoing any further treatment for injury or harm
- Thoughts/feelings about and willingness to seek future treatment in healthcare (e.g. psychological support, offer of further treatment, social or financial support, etc.)

1. **Summary** *(Aim: to round up the interview, safeguard and close)*

**Thank the participant and close**
